# Developing Resistance to Aflatoxin in Maize and Cottonseed

**DOI:** 10.3390/toxins3060678

**Published:** 2011-06-21

**Authors:** Jeffrey W. Cary, Kanniah Rajasekaran, Robert L. Brown, Meng Luo, Zhi-Yuan Chen, Deepak Bhatnagar

**Affiliations:** 1 United States Department of Agriculture-Agriculture Research Service, Southern Regional Research Center, New Orleans, LA 70124, USA; Email: rajah.rajasekaran@ars.usda.gov (K.R.); robert.brown@ars.udsa.gov (R.L.B.); meng.luo@ars.usda.gov (M.L.); deepak.bhatnagar@ars.usda.gov (D.B.); 2 Department of Plant Pathology and Crop Physiology, Louisiana State University Agricultural Center, Baton Rouge, LA 70803, USA; Email: zchen@agcenter.lsu.edu

**Keywords:** *Aspergillus flavus*, aflatoxin resistance, host resistance, marker-assisted breeding, transgenic cotton, maize

## Abstract

At this time, no “magic bullet” for solving the aflatoxin contamination problem in maize and cottonseed has been identified, so several strategies must be utilized simultaneously to ensure a healthy crop, free of aflatoxins. The most widely explored strategy for the control of aflatoxin contamination is the development of preharvest host resistance. This is because *A. flavus* infects and produces aflatoxins in susceptible crops prior to harvest. In maize production, the host resistance strategy has gained prominence because of advances in the identification of natural resistance traits. However, native resistance in maize to aflatoxin contamination is polygenic and complex and, therefore, markers need to be identified to facilitate the transfer of resistance traits into agronomically viable genetic backgrounds while limiting the transfer of undesirable traits. Unlike maize, there are no known cotton varieties that demonstrate enhanced resistance to *A. flavus* infection and aflatoxin contamination. For this reason, transgenic approaches are being undertaken in cotton that utilize genes encoding antifungal/anti-aflatoxin factors from maize and other sources to counter fungal infection and toxin production. This review will present information on preharvest control strategies that utilize both breeding and native resistance identification approaches in maize as well as transgenic approaches in cotton.

## 1. Introduction

Aflatoxins, are highly toxic and carcinogenic compounds produced by the fungi, *Aspergillus flavus *and* A. parasiticus *during growth on crops such as maize, peanut, cottonseed, and tree nuts [1]. *A. flavus* is most commonly associated with aflatoxin contamination of susceptible crops though *A. parasiticus* is often associated with contamination of peanut. The presence of aflatoxins in agricultural commodities poses a serious health threat to both humans and domesticated animals which is why the U.S. Food and Drug Administration (FDA) and their counterparts in many other countries have established strict regulations for aflatoxins in food and feeds [2]. The FDA has established an action level for total aflatoxins in human food at 20 parts per billion (ppb) and 0.5 ppb of aflatoxin M1 in milk. The European Union has enacted even stricter action levels for imported agricultural commodities. Action levels for aflatoxins have also been set for various categories of animal feed. Unfortunately, developing countries in many regions of the world, such as Sub-Saharan Africa, cannot afford the costs associated with monitoring and mitigation of aflatoxin in food and feed crops. This has led to an increased risk of exposure to aflatoxin resulting in outbreaks of acute aflatoxin poisoning (aflatoxicosis) [3] and increased morbidity in children suffering from stunted growth and malnutrition (kwashiorkor) [4,5]. In addition to the adverse effects that aflatoxin has on human and animal health worldwide there are also significant economic costs incurred trying to mitigate aflatoxin contamination of crops. Estimates reveal direct annual crop revenue losses in the U.S. in the tens of millions of dollars and in particularly severe years of Midwestern maize contamination losses can be in the hundreds of millions of dollars [6]. Of course total costs attributable to aflatoxin are much higher when you take into account crop losses in other countries in addition to other factors such as export market losses, sampling and testing, and human and animal health effects [7].

Pre-harvest aflatoxin contamination is a very complex problem affected by a multitude of biotic and abiotic factors. Therefore, a multi-pronged approach may need to be employed to control aflatoxin contamination when conditions in the field are favorable for fungal infection. An area of intense study for the control of aflatoxin contamination is the development of preharvest host plant-resistance [8,9]. This is because *A. flavus* infects affected crops prior to harvest and a host*-*resistance strategy may be the easiest for the grower to integrate into the various crop management systems to prevent preharvest contamination with aflatoxins. Several maize lines have been identified and developed with increased resistance to *A. flavus* infection and aflatoxin contamination and this has enabled the identification of natural resistance traits [10,11,12,13]. However, these investigations have indicated that resistance to aflatoxin contamination is polygenic. Therefore, attempts to move resistance from inbred lines into commercial varieties with desirable agronomic characteristics has been a slow process due to the lack of availability of biomarkers to facilitate the transfer of resistance genes [14]. Unlike maize, cotton has a limited diversity of germplasm and to date no varieties have been identified with natural resistance to *A. flavus*. For this reason, it is critical that a seed-based resistance be developed in cotton. A number of potential maize resistance-associated proteins (RAPs) and the genes encoding them have been identified and some of these have been introduced into cotton for evaluation [15,16,17]. However, more research is needed to elucidate the biochemical mechanisms that manifest the resistance phenotype in maize kernels or other sources so that they can be utilized to enhance resistance through marker*-*assisted breeding in maize or genetic engineering in cotton [12,18,19,20,21].

This review will focus on the following areas of research that will be critical for successful preharvest control of aflatoxin contamination in maize and cottonseed: (1) identification of new sources of maize germplasm resistant to fungal infection and aflatoxin contamination; (2) identification of RAPs and their corresponding genes in maize kernels through comparative proteomics and genomics of resistant and susceptible maize inbreds; (3) development of practical technology for use by maize breeders based upon identification of RAP*-*associated proteins (and genes) as resistance markers to aid in marker*-*assisted maize breeding; and (4) production of genetically engineered cotton with resistance alleles of RAP genes from maize and also genes from non*-*native sources to enhance resistance to aflatoxin contamination.

## 2. Host-Plant Resistance

### 2.1. Identification of Natural Resistance and Resistance Mechanisms in Maize

**Figure 1:**
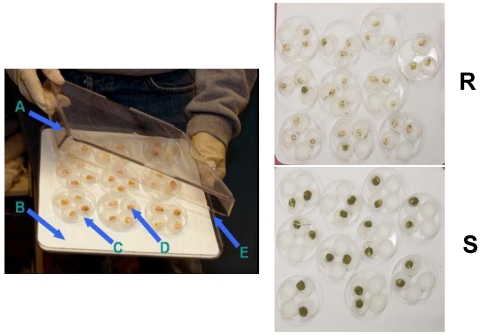
Kernel screening assay (KSA) apparatus illustrating the following features: Left panel: (**A**) bioassay tray lid; (**B**) chromatography paper for holding moisture; (**C**) Petri dish containing four kernels (experimental unit); (**D**) individual kernel in a vial cap, and (**E**) bioassay tray bottom. Right panels: Example of results from KSA experiment: R, resistant maize line; S, susceptible maize line.

There is a need to continually identify and utilize additional sources of corn genotypes with resistance to aflatoxin contamination. One contribution to the identification/evaluation of corn kernel resistance to aflatoxin contamination has been the development of a rapid laboratory screening assay. This assay, the kernel screening assay (KSA), was developed and used to study resistance to aflatoxin production in kernels of the maize breeding population, GT*-*MAS:gk (Figure 1) [22,23]. The KSA is designed to address the fact that aflatoxin buildup occurs in mature and not developing kernels. Although, other agronomic factors (e.g., husk tightness) are known to affect genotype resistance to aflatoxin accumulation in the field, the KSA measures seed*-*based resistance. The seed, of course, is the primary target of aflatoxigenic fungi, and is the edible portion of the crop. Therefore, seed*-*based resistance represents the core objective of corn host resistance. Towards this aim, the KSA has demonstrated proficiency in separating susceptible from resistant seed [22,23]. The results of KSA studies indicated the presence of two levels of resistance, at the pericarp and at the subpericarp level, since pin-wounding the pericarp led only to a partial loss in resistance in the GT-MAS:gk corn population. Significant expression of resistance was noted even in these wounded kernels, indicating a subpericarp source of resistance. Further studies demonstrated a role for pericarp waxes in kernel resistance [24,25,26] and highlighted quantitative and qualitative differences in pericarp wax between GT*-*MAS:gk and susceptible genotypes [26,27]. The KSA confirmed sources of resistance among 31 inbreds tested in Illinois field trials [13,23], thus demonstrating that the KSA can be used, at least initially, to rank corn for its field resistance to aflatoxin contamination. 

The KSA also identified potentially aflatoxin*-*resistant corn germplasm among inbred lines selected in West and Central Africa for ear*-*rot resistance, for inclusion as parents in an International Institute of Tropical Agriculture (IITA)-Southern Regional Research Center (SRRC) aflatoxin*-*resistance collaborative breeding program [28,29]. This program’s objective is to combine resistance of lines selected in Central and West Africa for ear rot resistance to one to several fungi (including *A. flavus* and *Fusarium verticillioides*, a fumonisin producer) with resistance in inbred lines from the U.S. in order to develop improved resistant lines with desirable agronomic traits useful in U.S. breeding programs and in national programs of Central and West Africa. From the S_5_ generation on, the KSA was used to screen progeny and decide which lines would be moved to the field for testing in Nigeria. Recently, six inbred lines with aflatoxin-resistance, in good agronomic backgrounds were registered and released in the U.S. by this program for further testing and development towards commercialization [14]. The KSA has demonstrated several advantages, as compared to traditional field screening techniques [23]: (1) it can be performed and repeated several times throughout the year and outside of the growing season; (2) it requires few kernels; (3) it can detect/identify different kernel resistance mechanisms; (4) it can dispute or confirm field evaluations (identify escapes); and (5) correlations between laboratory findings and inoculations in the field have been demonstrated. The KSA can, therefore, be a valuable complement to standard breeding practices for preliminary evaluation of germplasm. However, field trials are necessary for the final confirmation of resistance.

### 2.2. The Use of Reporter Genes in Maize Germplasm Evaluations

Three resistant inbreds (MI82, CI2, and T115) were examined among the 31 tested in Illinois field trials, using a modified KSA, which included an *A. flavus* GUS transformant strain genetically engineered with a β*-*glucuronidase reporter gene linked to an *A. flavus* β*-*tubulin gene promoter for monitoring fungal growth. Our results demonstrated, both visually and quantitatively, kernel resistance to fungal infection in non-wounded and wounded kernels, and a statistically significant positive relationship between the degree of fungal infection and aflatoxin levels [23,30]. However, in the KSA investigation of West and Central African lines, growth of the *A. flavus* GUS transformant and aflatoxin accumulation did not always correlate positively [28]. This opens the possibility of identifying resistance mechanisms that inhibit aflatoxin biosynthesis rather than fungal growth, adding to the variety of traits that could be transferred into agronomically useful germplasm to control aflatoxin contamination. 

*A. flavus* transformants with the GUS reporter gene linked to an aflatoxin biosynthetic pathway gene could also provide a quick and economical way to indirectly measure aflatoxin levels [31,32], based on the level of expression of the GUS gene. The utility of improved tester strains of *A. flavus* expressing the GFP reporter gene has now been shown in corn [33] and in cotton [34].

## 3.Identification of Resistance-Associated Proteins (RAPs) in Maize

Studies demonstrating subpericarp (wounded*-*kernel) resistance in corn kernels have led to research for identification of subpericarp resistance mechanisms. The examination of kernel proteins of several genotypes revealed differences between resistant and susceptible genotypes [35]. The first proteins shown to be potentially involved in corn kernel resistance were germination*-*induced ribosome inactivating protein (RIP) and zeamatin [36], which were also shown to be involved in inhibition *in vitro* of *A. flavus* growth. It has also been shown that both constitutive and induced kernel proteins are necessary for the expression of kernel resistance to aflatoxin production [37], and that a high level of expression of constitutive antifungal proteins actually constitutes a major difference between resistant and susceptible kernels. 

In a study of protein production in corn inbred Tex6, two kernel proteins were identified which may contribute to its resistance to aflatoxin production [38]. One of the proteins, 28 kDa in size, inhibited *A. flavus* growth, while a second with a molecular mass greater than 100 kDa, inhibited toxin formation with little effect on fungal growth. The 28 kDa protein was identified as a unique chitinase [39]. In another investigation, an examination of kernel protein profiles of 13 corn genotypes revealed that a 14 kDa trypsin inhibitor protein (TI) is present at relatively high concentrations in seven resistant corn lines, but is present only in low concentrations in six susceptible ones [40]. The mode of action of TI against fungal growth may be partially due to its inhibition of fungal α*-*amylase [41], limiting *A. flavus* access to potential simple sugars required for toxin production [42]. Comparisons of kernel protein profiles between susceptible and resistant genotypes may shorten the time it takes to identify RAPs. 

Proteomics can also enhance the identification of RAPs. Through proteomic analysis and side*-*by*-*side comparisons of constitutive kernel embryo and endosperm proteins of resistant and susceptible genotypes, unique or elevated levels of stress*-*related proteins were discovered in aflatoxin*-*resistant lines [16,43]. These proteins can be grouped into three categories based on their peptide sequence homology: (1) storage proteins, such as globulins (GLB1, GLB2), and late embryogenesis abundant proteins (LEA3, LEA14); (2) stress-responsive proteins, such as aldose reductase (ALD), glyoxalase I (GLX1) and heat shock proteins, and (3) antifungal proteins, including TI. The discovery of stress-related RAPs may be very important given the observation of drought’s enhancement of aflatoxin accumulation. 

Another valuable contribution to RAP identification has been the discovery of closely-related lines from the same backcross differing in aflatoxin accumulation for proteomic analysis in the IITA-SRRC collaborative breeding program [28,29]. Investigating corn lines sharing close genetic backgrounds should enhance the identification of RAPs without the confounding effects experienced with lines of diverse genetic backgrounds [12]. Using pairs of closely-related lines, a proteomic study was conducted which confirmed the earlier identification of three categories of proteins and added a fourth category, putative regulatory proteins [44]. Regulatory proteins, not always easily seen on 2-D gels, may have been more identifiable due to the use of closely-related lines. Proteomics has been used to study corn resistance using rachis and silk tissue, as well [45,46]. Results, as might be expected, support the findings of kernel studies regarding genotypic differences in resistance. 

### Further Characterization of RAPs towards Use as Markers

Of the constitutively-expressed RAPs that have been identified, several have been further investigated to understand their potential involvement in resistance. Among these are: (1) aldose reductase (ALD), (2) glyoxalase I (GLX-I), (3) pathogenesis related protein 10 (PR-10), (4) peroxiredoxin antioxidant (PER1), (5) cold-regulated-like protein (ZmCOR), (6) trypsin inhibitor, ZmTI, and 14 kDa TI [12]. 

Aldose reductase, reported to have a role in stress tolerance, is produced constitutively at higher levels in kernel embryo tissue of resistant *versus* susceptible maize genotypes [43]. Glyoxalase I, produced in the kernel embryo, is involved in the conversion of cytotoxic methylglyoxal (MG) into D-lactate, along with GLX II, and is suggested to be important to plant stress tolerance [47]. Higher GLX-I activity was observed in maize kernels of resistant genotypes than in susceptibles both constitutively and after *A. flavus* infection. However, infection significantly increased MG levels in two of three susceptible lines. MG was also shown to induce aflatoxin production *in vitro* [47]. During an investigation of PR-10, which is produced in kernel endosperm [17], it was discovered that *PR10 *expression increased fivefold between 7 and 22 days after pollination, and was induced upon *A. flavus* infection in the resistant but not the susceptible genotype. It was also shown that PR-10 had ribonucleolytic and antifungal activities against *A. flavus*. RNAi-induced silencing of *PR10* expression indicated an important role for PR-10 in aflatoxin-resistance [15]. A new *PR10* homologue, *PR10.1*, was identified from maize [48]. *PR10 *was expressed at higher levels in all tissues compared to *PR10.1*, however, purified PR-10.1 overexpressed in *E. coli *possessed 8-fold higher specific RNase activity than PR-10*. *This homologue may also play a role in resistance.

PER1 protein, also produced in the endosperm, demonstrated peroxidase activity *in vitro*, and *PER1* expression during late development was significantly higher in a resistant *versus* the susceptible genotype, and was significantly induced upon *A. flavus* infection [16]. ZmTIp, a 10 kDa trypsin inhibitor, had an impact on fungal growth, but not as great as previously investigated TIs [49]. The roles of GLX I and the 14 kDa TI are currently being evaluated by RNAi-induced gene silencing methods. 

## 4. Plant Molecular Breeding Strategies

Chromosome regions associated with resistance to *A. flavus* and inhibition of aflatoxin production in maize have been identified through Restriction Fragment Length Polymorphism (RFLP) analysis in three “resistant” lines (R001, LB31, and Tex6) in an Illinois breeding program, after mapping populations were developed using B73 and/or Mo17 elite inbreds as the “susceptible” parents [50,51]. Chromosome regions associated with inhibition of aflatoxin included regions on chromosome arms 2L, 3L, 4S, and 8S and these may prove promising for improving resistance through marker-assisted breeding into commercial lines. In some cases, chromosomal regions were associated with resistance to *Aspergillus* ear rot and not aflatoxin inhibition, and vice versa, whereas other chromosomal regions were found to be associated with both traits. This suggests that these two traits may be at least partially under separate genetic control.

Quantitative trait locus (QTL) studies involving other populations have identified chromosome regions associated with low aflatoxin accumulation. In a study involving 2 populations from Tex6 × B73, promising QTLs for low aflatoxin were detected in bins 3.05–3.06, 4.07–4.08, 5.01–5.02, 5.05–5.05, and 10.05–10.07 [52]. Environment strongly influenced detection of QTLs for lower toxin in different years; QTLs for lower aflatoxin were attributed to both parental sources. In a study involving a cross between B73 and resistant inbred Oh516, QTL associated with reduced aflatoxin were identified on chromosomes 2, 3, and 7 (bins 2.01 to 2.03, 2.08–2.09, 3.08–3.09, and 7.06–7.07) [53]. QTLs contributing resistance to aflatoxin accumulation were also identified using a population created by B73 and resistant inbred Mp313E, on chromosome 4 of Mp313E [54]. Another QTL in this study, which has similar effects to that on chromosome 4, was identified on chromosome 2. In recent studies to identify aflatoxin-resistance QTL and linked markers for marker-assisted breeding (using a population developed from an aflatoxin-resistant maize inbred, Mp717 and a susceptible NC300), QTL were identified on all chromosomes, except 4, 6, and 9 [55], and on chromosomes 1,3,5, and 10 in multiple years (4 and 9 in one year) when using a population developed using resistant inbred Mp715 and susceptible T713 [56]. In all of the above-mapping projects, no QTL was identified that contributed more than ~20% to phenotypic variation [57].

A number of RAP genes identified through comparative proteomics have been mapped to chromosomal location [12] using the genetic sequence of B73 now available online [57]. Using the DNA sequence of the RAPs and running a BLAST similarity search against the B73 sequence allowed each gene to be placed into a virtual Bin, facilitating the mapping of their exact location on the chromosome. The chromosomes involved include the above-mentioned chromosomes 1, 2, 3, 7, 8, and 10, and some in bins closely located to those described above. This adds support to proteomic data and characterization results that suggest the involvement of the 14 kDa TI, water stress inducible protein, zeamatin, one of the heat shock proteins, a cold-regulated protein, glyoxalase I, PR-10 protein and beta-1-3-glucanase in aflatoxin-resistance. In fact, heat shock protein 16.9 mapped with a QTL (bin 3.04–3.05) that accounted for a relatively high level (16%) of phenotypic variation [45]. From the above QTL investigations, it is observed that variation can exist in the chromosomal regions associated with *Aspergillus* ear rot and aflatoxin inhibition in different mapping populations. This suggests the presence of different genes for resistance in the different identified resistant germplasm. It will be important to map resistant lines investigated through proteomics or to obtain data from associative mapping panels regarding gene location.

## 5. Genetic Engineering Strategies

Conventional breeding in maize has delivered much-improved cultivated crops through enhancement of agronomic traits such as disease and stress resistance. These successes have been possible because of the availability of desired resistance genes in the maize germplasm [8,12,58]. A genetic engineering approach in cotton is warranted as natural resistance to mycotoxin-producing, saprophytic fungal pathogens such as *A. flavus* has not been identified in the germplasm base. For this reason, transgenic cotton varieties with antifungal traits that confer resistance to *A. flavus* will be extremely valuable. Available literature on transgenic crops exhibiting microbial resistance is mostly on bacterial or viral resistance. In fact, no fungal resistant crops have yet been deregulated in spite of several field tests, underlying the complexity of host-pathogen relationships. This gets more confounded by the fact that *A. flavus* is a saprophyte, and not a typical plant pathogen that demonstrates a gene for gene relationship. Success of a genetic engineering approach in developing cotton with increased resistance to *A.flavus* infection and aflatoxin contamination will depend in large part on identification of resistance genes, native or foreign, with target specificity that express inhibitory activity against aflatoxigenic fungi [59,60,61]. Table 1 summarizes information on a number of candidate proteins/peptides from maize and other sources that have been tested for efficacy against *A. flavus* as well as other fungal plant pathogens.

**Table 1:** Natural and synthetic proteins/peptides with antifungal activity against *Aspergillus flavus*.

Protein/Peptide	Protein Family	Source	Mode of Action	Reference
Haloperoxidase	peroxidase	*Pseudomonas pyrrocinia *	produce antimicrobial compounds - peracetic acid and hypohalites	[62,63]
β-1-3 glucanase	glycosyl hydrolase	tobacco	hydrolysis of fungal cell wall components	[64]
Ib-AMP_3_	defensin	sweet potato	lytic	[65]
AILp	lectin	hyacinth bean	inhibits germination and hyphal growth	[66]
Chitinase	glycosyl hydrolase	corn inbred Tex6	hydrolysis of fungal cell wall components	[39]
ZmCORp	lectin	corn kernels	hemagglutination activity against fungal conidia	[67]
Mod-1/RIP-1	ribosome-inhibiting protein	corn	inhibits hyphal tip growth	[68,69]
Zeamatin	PR-5	corn	inhibits hyphal tip growth	[36]
ZmPR-10	PR-10	corn	RNAse activity	[17]
Trypsin inhibitor	protease inhibitor	corn	trypsin/amylase inhibition	[40]
Purothionin hordothionin	thionin	barley wheat	lytic	Rajasekaran unpublished
D4E1		synthetic peptide	lytic	[70,71]
D5C/D5C1		synthetic peptide	lytic	[72]
D2A21		synthetic peptide	lytic	[72]
MSI99		synthetic peptide	lytic	[73]

### 5.1. Candidate Genes from Maize

Many endogenous low molecular weight compounds and bio-macromolecules in kernel tissues have been identified as antifungal at various stages of kernel development in grain crops [11,12,74,75,76,77]. However, compounds with activity against other fungal species are often ineffective against *A. flavus*, and thus it is important to select the best candidate inhibitory compounds and identify and characterize their respective genes before plant genetic engineering procedures are initiated. A list of candidate antifungal compounds may include RIPs, lectins, relatively small molecular weight (MW) polypeptides, cell*-*surface glycoproteins, hydrolases, and certain basic proteins [12,16,17]. A number of RAPs have been identified through comparative proteomics of closely-related maize lines that vary in aflatoxin accumulation (see Section 3; reviewed in [12]). For example, trypsin inhibitor (TI) was shown to be correlated with kernel resistance to *A. flavus* infection of maize [40]. Evidence from several IITA maize genotypes (progeny of U.S. and African parental lines), resistant to preharvest aflatoxin contamination, indicates that TI plays a key role in imparting this resistance. When cotton was transformed with the *TI *gene under the control of the enhanced double CaMV 35S promoter, the expression level was sufficient to control *Verticillium* but not *A. flavus* [78], indicating a need for higher seed-specific expression of this gene in cottonseed. 

A number of other maize kernel proteins potentially inhibitory to *A. flavus* and aflatoxin accumulation have been evaluated (see Section 3 and Table 1). These include PR*-*10, a pathogenesis related protein with antifungal and RNase activity [15,79] and glyoxalase I [47], a stress*-*related protein with demonstrated potential to directly inhibit aflatoxin accumulation. RIP-1 is a ribosome inactivating protein from maize that has been shown to exhibit useful levels of antifungal activity against *A. nidulans in vitro* and is thought to be associated with observed control of *A. flavus *growth in resistant maize lines [68,80]. RIP-1 has negligible toxicity to humans and very low activity against ribosomes of maize and other plant species [81]. *Mod1* is a synthetic gene that encodes the proteolytically-activated form of RIP-1 [81]. Transgenic peanut expressing *MOD1* have demonstrated increased resistance to *A. flavus* growth and aflatoxin contamination in detached cotyledon assays [69]. The maize kernel PR proteins appear to have a function during the normal process of seed germination [17,75,82]. It appears that they are induced to accumulate in response to fungal infection, and their expression is tissue*-*specific [82,83]. A chitinase isolated from the maize inbred Tex6 was shown to inhibit the growth of *A. flavus* by 50% at a concentration of 20 μg/mL, however the gene encoding the chitinase has yet to be cloned [39]. Proteomics of maize kernel proteins also identified a protein, ZmCORp, that was present at higher levels in a resistant line of maize compared to levels in a sensitive line [67]. The protein demonstrated homology to cold-regulated proteins and recombinant ZmCORp exhibited lectin-like hemagglutination activity against fungal conidia and sheep erythrocytes. ZmCORp exhibited fungistatic activity when conidia from *A. flavus *were exposed to the protein at a final concentration of 18 mM. ZmCORp inhibited the germination of *A. flavus* conidia by 80% and a 50% decrease in mycelial growth was observed when germinated conidia were incubated with the protein.

### 5.2. Candidate Genes from Other Sources

A number of potentially useful antifungal enzymes/proteins are produced either constitutively or in response to fungal attack in plants (reviewed in [61,84,85,86]). These include chitinases and β*-*1,3*-*glucanases, osmotins, protease inhibitors and even regulatory proteins such as the defense response protein, NPR1, from Arabidopsis [87]. Additionally, small antimicrobial peptides (a peptide is often defined as a small protein of less than 40 amino acids), important components of non-specific host defense systems and innate immunity in insects, amphibians, plants, and mammals, have been the subject of numerous studies to enhance host plant resistance to bacterial and fungal pathogens (reviewed in [88,89,90,91,92]). Several of these natural peptides possess nonspecific toxicity to non-target organisms and are subject to proteolytic degradation. The advent of automated peptide synthesizers and combinatorial peptide chemistry over the past decade has made it possible for rational synthesis of stable and target-specific peptides to overcome some of the problems associated with natural lytic peptides (reviewed in [88]). Transgenic plants expressing genes for synthetic analogs of cecropins and magainins have demonstrated improved resistance to fungal invasion [70,73]. For the purpose of this review, the information presented is limited to proteins/peptides from sources other than maize that have been analyzed for activity against *A. flavus*.

Certain small lytic peptides have demonstrated convincing inhibitory activity against *A. flavus* and show promise for transformation of plants to reduce infection of seed. A synthetic lytic peptide (*D4E1*) gene, when transformed into tobacco, greatly enhanced resistance to *C. destructivum in planta* [70,93,94]. In addition to inhibiting the germination of *A. flavus* spores, D4E1, at concentrations of 10–25 µM caused severe abnormal lytic effects on mycelial wall, cytoplasm, and nuclei in *in vitro* studies. In tests with cottonseed from plants transformed with the *D4E1* gene, resistance to penetration of cottonseed coats by a GFP reporter gene*-*expressing *A. flavus* strain was observed [34,71]. The expression of *D4E1* was sufficient enough to inhibit the growth *in vitro* of *Fusarium verticillioides* and *Verticillium dahliae* or *in planta* of* Thielaviopsis basicola* [71]. The antimicrobial peptide MSI*–*99, an analog of magainin 2, was expressed via the chloroplast genome of tobacco [73]. Leaf extracts from T1 generation plants inhibited the growth of pre-germinated spores of three fungal species, *A. flavus*, *Fusarium verticillioides*, and *Verticillium dahliae*, by more than 95%, compared with non*–*transformed control plant extracts. The levels of MSI-99 peptide in the extracts were not determined.

Antifungal activities of thionin have been described previously [95,96]. Thionins are low-molecular weight proteins that are believed to exert their antimicrobial properties via an electrostatic interaction with the negatively charged phospholipids of the fungal membrane resulting in pore formation and eventual cell death [95]. Studies have determined that pre*-*germinated spores of *A. flavus* were fully inhibited from further development by purothionin or hordothionin at about 10 µM (Rajasekaran *et al*., unpublished data). A nonheme chloroperoxidase gene (*cpo-p*) from *Pseudomonas pyrrocinia* was introduced into peanut via particle bombardment. *In vitro* bioassay using crude protein extracts from transgenic T0, T1, and T4 plants showed inhibition of *Aspergillus flavus* hyphal growth [97]. A 36-kDa protein isolated from *Lablab purpureus*, denoted AILp, has been shown to inhibit alpha-amylase production and the growth of *A. flavus *[98]. Expression of the *LABAI-1* and *LABAI-2* genes from *L. purpureus* in a yeast expression system yielded recombinant proteins that demonstrated agglutination of human red blood cells and inhibited *A. flavus* alpha-amylase in a manner similar to that shown by AILp. These data indicate that *LABAI* genes are a new class of lectin members in legume seeds and that their proteins have both lectin and alpha-amylase inhibitor activity.

## 6. Conclusions

Outbreaks of severe aflatoxin contamination of maize and cottonseed are inevitable and appear to be caused in large part by stress on the host plant usually in the form of drought and/or insect pressure. It is likely that significant control of aflatoxin contamination will require a multipronged approach that utilizes biological control and improved agronomic practices as well increased resistance by the host plant arising from either marker-assisted breeding in maize or genetic engineering of cotton. To this end, the identification of natural resistance traits to aflatoxin accumulation in maize genotypes has provided an inroad to the development of a host resistance strategy in which genes encoding resistance associated proteins can be utilized as molecular markers for transfer of aflatoxin resistance traits into elite maize varieties. Much work has been accomplished on the identification of maize genotypes demonstrating increased resistance to aflatoxin contamination and now technologies such as proteomics and genomics are being utilized to identify the proteins/genes that contribute to the observed resistance. Of equal importance in selecting candidate resistance-associated genes will be to determine what effects stress has on their expression and at what developmental stage and in what tissues they are being expressed so as to maximize their efficacy against *A. flavus*. 

Development of cotton with enhanced resistance to aflatoxin contamination will be more problematic. The lack of genetic diversity in the germplasm renders conventional breeding approaches challenging as no natural resistance to aflatoxin accumulation has been identified in cotton. This is why it is imperative that genes encoding resistance-associated proteins from maize be identified and introduced into cotton by transgenic approaches in an effort to control *A. flavus* growth and aflatoxin production. Knowing that resistance in maize is multigenic it is very likely that improved resistance in cotton will require engineering of cotton with multiple resistance genes. However, sources of resistance for development of transgenic cottons do not have to originate solely from maize and this review has presented information on the efficacy of a number of potential antifungal proteins/peptides from sources other than maize against *A. flavus. *Of particular interest are the small, lytic, antifungal peptides, both natural and synthetic. Utilizing automated peptide synthesis chemistry it is relatively simple to have a peptide synthesized for use in *in vitro* assays against *A. flavus*. If necessary, promising natural antifungal peptides can be modified to increase their potency and specificity toward *A. flavus* utilizing rational design approaches. Because many of these antifungal peptides act via membrane permeabilization, there is less likelihood that *A. flavus* or other pathogens will be able to develop resistance to these peptides. Bioassays in our labs have indicated that *A. flavus* usually requires the highest level of antifungal protein/peptide concentrations to inhibit its growth compared to other fungal pathogens. Therefore, identification of a single or combination of genes encoding protein/peptides that control *A. flavus *growth when expressed in maize or cotton will probably be effective against a number of fungal pathogens that infect food and feed crops.

Another, as of yet unexplored, transgenic approach for aflatoxin resistance in maize and cotton is the use of RNA interference (RNAi) as a means of downregulating expression of genes vital to fungal growth and toxin formation. In this scenario, the transgenic plant would be engineered with vectors for the expression of self-complementary hairpin RNAs of antifungal/anti-aflatoxin genes that will result in production of small interfering RNAs (siRNAs) by the host plant’s DICER-like enzymes. This technique has been successfully demonstrated *in planta* for silencing of *gus* gene expression in *Fusarium verticillioides* interacting with transgenic tobacco generating *gus* siRNAs [99]. Critical to the success of this approach will be the ability of the invading fungus to efficiently take up the host-plant generated siRNAs *in planta* to activate the machinery for silencing of the targeted gene. 

## References

[1] Center for Applied Special Technology (1979). Aflatoxins and Other Mycotoxins: An Agricultural Perspective.

[2] Food and Agriculture Organization (2004). Worldwide Regulations for Mycotoxins in Food and Feed in 2003.

[3] Probst C., Schulthess F., Cotty P.J. (2009). Impact of *Aspergillus* section *Flavi* community structure on the development of lethal levels of aflatoxins in Kenyan maize (*Zea mays*). J. Appl. Microbiol..

[4] Turner P.C., Moore S.E., Hall A.J., Prentice A.M., Wild C.P. (2003). Modification of immune function through exposure to dietary aflatoxin in Gambian children. Environ. Health Perspect..

[5] Gong Y.Y., Hounsa A., Egal S., Turner P.C., Sutcliffe A.E., Hall A.J., Cardwell K., Wild C.P. (2004). Postweaning exposure to aflatoxin results in impaired child growth: A longitudinal study in Benin, West Africa. Environ. Health Perspect..

[6] Richard J.A., Payne G.A. (2003). Mycotoxins: Risk in Plant, Animal, and Human Systems.

[7] Wu F. (2004). Mycotoxin risk assessment for the purpose of setting international regulatory standards. Environ. Sci. Technol..

[8] Bhatnagar D., Rajasekaran K., Cary J.W., Brown R.L., Yu J., Cleveland T.E. (2008). Molecular Approaches to Development of Resistance to Preharvest Aflatoxin Contamination. Mycotoxins: Detection Methods, Management, Public Health and Agricultural Trade.

[9] Cleveland T.E., Dowd P.F., Desjardins A.E., Bhatnagar D., Cotty P.J. (2003). United States department of agriculture-agricultural research service research on pre-harvest prevention of mycotoxins and mycotoxigenic fungi in US crops. Pest Manag. Sci..

[10] Brown R.L., Chen Z.Y., Menkir A., Cleveland T.E. (2006). Proteomics to identify resistance factors in corn—a review. Mycotoxin Res..

[11] Brown R.L., Chen Z.Y., Gembeh S.V., Cleveland T.E., Bhatnagar D., Howard K. (2004). Identification of natural resistance in corn against mycotoxin-producing fungi. Rec. Adv. Food Sci..

[12] Brown R.L., Chen Z.Y., Warburton M., Luo M., Menkir A., Fakhoury A., Bhatnagar D. (2010). Discovery and characterization of proteins associated with aflatoxin-resistance: Evaluating their potential as breeding markers. Toxins.

[13] Campbell K.W., White D.G. (1995). Evaluation of corn genotypes for resistance to *Aspergillus* ear rot, kernel infection, and aflatoxin production. Plant Dis..

[14] Menkir A., Brown R.L., Bandyopadhyay R., Cleveland T.E. (2008). Registration of six tropical maize germplasm lines with resistance to aflatoxin contamination. J. Plant Regist..

[15] Chen Z.Y., Brown R.L., Damann K.E., Cleveland T.E. (2010). PR10 expression in maize and its effect on host resistance against *Aspergillus flavus* infection and aflatoxin production. Mol. Plant Pathol..

[16] Chen Z.Y., Brown R.L., Damann K.E., Cleveland T.E. (2007). Identification of maize kernel endosperm proteins associated with resistance to aflatoxin contamination by *Aspergillus flavus*. Phytopathology.

[17] Chen Z.Y., Brown R.L., Rajasekaran K., Damann K.E., Cleveland T.E. (2006). Identification of a maize kernel pathogenesis-related protein and evidence for its involvement in resistance to *Aspergillus flavus* infection and aflatoxin production. Phytopathology.

[18] Cary J.W., Rajasekaran K., Yu J., Brown R.L., Bhatnagar D., Cleveland T.E. (2009). Transgenic approaches for pre-harvest control of mycotoxin contamination in crop plants. World Mycotoxin J..

[19] Rajasekaran K., Jaynes J.M., Cary J.W., Appell M., Kendra D.F., Trucksess M.W. (2009). Transgenic Expression of Lytic Peptides in Food and Feed Crops to Control Phytopathogens and Preharvest Mycotoxin Contamination. Mycotoxin Prevention and Control in Agriculture.

[20] Rajasekaran K., Cary J.W., Cleveland T.E. (2006). Prevention of preharvest aflatoxin contamination through genetic engineering of crops. Mycotoxin Res..

[21] Brown R.L., Chen Z.Y., Cleveland T.E., Menkir A., Fakhoury A., Appell M., Kendra D.F., Trucksess M.W. (2009). Identification of Maize Breeding Markers through Investigations of Proteins Associated with Aflatoxin-Resistance. Mycotoxin Prevention and Control in Agriculture.

[22] Brown R.L., Cotty P.J., Cleveland T.E., Widstrom N.W. (1993). Living maize embryo influences accumulation of aflatoxin in maize kernels. J. Food Prot..

[23] Brown R.L., Cleveland T.E., Payne G.A., Woloshuk C.P., Campbell K.W., White D.G. (1995). Determination of resistance to aflatoxin production in maize kernels and detection of fungal colonization using an *Aspergillus flavus* transformant expressing *Escherichia coli* β-glucuronidase. Phytopathology.

[24] Guo B.Z., Russin J.S., Cleveland T.E., Brown R.L., Widstrom N.W. (1995). Wax and cutin layers in maize kernels associated with resistance to aflatoxin production by *Aspergillus flavus*. J. Food Prot..

[25] Guo B.Z., Russin J.S., Cleveland T.E., Brown R.L., Damann K.E. (1996). Evidence for cutinase production by *Aspergillus flavus* and its possible role in infection of corn kernels. Phytopathology.

[26] Gembeh S.V., Brown R.L., Grimm C., Cleveland T.E. (2001). Identification of chemical components of corn kernel pericarp wax associated with resistance to *Aspergillus flavus* infection and aflatoxin production. J. Agric. Food Chem..

[27] Russin J.S., Guo B.Z., Tubajika K.M., Brown R.L., Cleveland T.E., Widstrom N.W. (1997). Comparison of kernel wax from corn genotypes resistant or susceptible to *Aspergillus flavus*. Phytopathology.

[28] Brown R.L., Chen Z.Y., Menkir A., Cleveland T.E., Cardwell K., Kling J., White D.G. (2001). Resistance to aflatoxin accumulation in kernels of maize inbreds selected for ear rot resistance in West and Central Africa. J. Food Prot..

[29] Menkir A., Brown R.L., Bandyopadhyay R., Chen Z.Y., Cleveland T.E. (2006). A U.S.A.-Africa collaborative strategy for identifying, characterizing, and developing maize germplasm with resistance to aflatoxin contamination. Mycopathologia.

[30] Brown R.L., Cleveland T.E., Payne G.A., Woloshuk C.P., White D.G. (1997). Growth of an *Aspergillus flavus* transformant expressing *Escherichia coli* β-glucuronidase in maize kernels resistant to aflatoxin production. J. Food Prot..

[31] Payne G.A. Characterization of Inhibitors from Corn Seeds and the Use of a New Reporter Construct to Select Corn Genotypes Resistant to Aflatoxin Accumulation. Proceedings of the 10th USDA ARS Aflatoxin Elimination Workshop.

[32] Brown R.L., Brown J.C.S., Bhatnagar D., Payne G.A. (2003). Construction and preliminary evaluation of an *Aspergillus flavus* reporter gene construct as a potential tool for screening aflatoxin resistance. J. Food Prot..

[33] Du W., Huang Z., Flaherty J.E., Wells K., Payne G.A. (1999). Green fluorescent protein (GFP) as a reporter to monitor gene expression and colonization by *Aspergillus flavus*. Appl. Environ. Microbiol..

[34] Rajasekaran K., Cary J.W., Cotty P.J., Cleveland T.E. (2008). Development of a GFP-expressing *Aspergillus flavus* strain to study fungal invasion, colonization, and resistance in cottonseed. Mycopathologia.

[35] Guo B.Z., Brown R.L., Lax A.R., Cleveland T.E., Russin J.S., Widstrom N.W. (1998). Protein profiles and antifungal activities of kernel extracts from corn genotypes resistant and susceptible to *Aspergillus flavus*. J. Food Prot..

[36] Guo B.Z., Chen Z.Y., Brown R.L., Lax A.R., Cleveland T.E., Russin J.S., Mehta A.D., Selitrennikoff C.P., Widstrom N.W. (1997). Germination induces accumulation of specific proteins and antifungal activities in corn kernels. Phytopathology.

[37] Chen Z.Y., Brown R.L., Cleveland T.E., Damann K.F., Russin J.S. (2001). Comparison of constitutive and inducible maize kernel proteins of genotypes resistant or susceptible to aflatoxin production. J. Food Prot..

[38] Huang Z., White D.G., Payne G.A. (1997). Corn seed proteins inhibitory to *Aspergillus flavus* and aflatoxin biosynthesis. Phytopathology.

[39] Moore K.G., Price M.S., Boston R.S., Weissinger A.K., Payne G.A. (2004). A chitinase from Tex6 maize kernels inhibits growth of *Aspergillus flavus*. Phytopathology.

[40] Chen Z.Y., Brown R.L., Lax A.R., Guo B.Z., Cleveland T.E., Russin J.S. (1998). Resistance to *Aspergillus flavus* in corn kernels is associated with a 14-kDa protein. Phytopathology.

[41] Chen Z.Y., Brown R.L., Russin J.S., Lax A.R., Cleveland T.E. (1999). A corn trypsin inhibitor with antifungal activity inhibits *Aspergillus flavus* α-amylase. Phytopathology.

[42] Woloshuk C.P., Cavaletto J.R., Cleveland T.E. (1997). Inducers of aflatoxin biosynthesis from colonized maize kernels are generated by an amylase activity from *Aspergillus flavus*. Phytopathology.

[43] Chen Z.Y., Brown R.L., Damann K.E., Cleveland T.E. (2002). Identification of unique or elevated levels of kernel proteins in aflatoxin-resistant maize genotypes through proteome analysis. Phytopathology.

[44] Chen Z.Y., Brown R.L., Menkir A., Cleveland T.E. (2011). Identification of resistance-associated proteins in closely-related maize lines varying in aflatoxin accumulation. Mol. Breed..

[45] Pechanova O. (2006). Proteomic analysis of maize rachis from inbred lines resistant and susceptible to *Aspergillus flavus*.

[46] Peethambaran B., Hawkins L., Windham G.L., Williams W.P., Luthe D.S. (2010). Antifungal activity of maize silk proteins and role of chitinases in *Aspergillus flavus* resistance. Toxin Rev..

[47] Chen Z.Y., Brown R.L., Damann K.E., Cleveland T.E. (2004). Identification of a maize kernel stress-related protein and its effect on aflatoxin accumulation. Phytopathology.

[48] Xie Y.R., Chen Z.Y., Brown R.L., Bhatnagar D. (2010). Expression and functional characterization of two pathogenesis-related protein 10 genes from *Zea mays*. J. Plant Physiol..

[49] Baker R., Brown R.L., Cleveland T.E., Chen Z.Y., Fakhoury A. (2009). A maize trypsin inhibitor (ZmTIp) with limited activity against *Aspergillus flavus*. J. Food Prot..

[50] White D.G., Rocheford T.R., Kaufman B., Hamblin A.M. Chromosome Regions Associated with Resistance to *Aspergillus flavus* and Inhibition of Aflatoxin Production in Maize. Proceedings of the 8th USDA ARS Aflatoxin Elimination Workshop.

[51] White D.G., Rocheford T.R., Naidoo G., Paul C., Hamblin A.M. Inheritance of Molecular Markers Associated with, and Breeding for Resistance to *Aspergillus* Ear Rot and Aflatoxin Production in Corn Using Tex6. Proceedings of the 11th USDA-ARS Aflatoxin Elimination Workshop.

[52] Paul C., Naidoo G., Forbes A., Mikkilineni V., White D., Rocheford T. (2003). Quantitative trait loci for low aflatoxin production in two related maize populations. Theor. Appl. Genet..

[53] Busboom K.N., White D.G. (2004). Inheritance of resistance to aflatoxin production and *Aspergillus* ear rot of corn from the cross of inbreds B73 and Oh516. Phytopathology.

[54] Brooks T.D., Williams W.P., Windham G.L., Wilcox M.C., Abbas H. (2005). Quantitative trait loci contributing resistance to aflatoxin accumulation in maize inbred Mp313E. Crop Sci..

[55] Warburton M.L., Brooks T.D., Krakowsky M.D., Shan X., Windham G.L., Williams W.P. (2009). Identification and mapping of new sources of resistance to aflatoxin accumulation in maize. Crop Sci..

[56] Warburton M.L., Brooks T.D., Windham G.L., Williams W.P. (2010). Identification of novel QTL contributing resistance to aflatoxin accumulation in maize. Mol. Breed..

[57] Maize Sequence. http://archive.maizesequence.org/index.html.

[58] Center for Applied Special Technology (2003). Aflatoxins and other Mycotoxins: An Agricultural Perspective.

[59] Stewart J.M., Nader C.A., Rajasekaran K. (2007). Effect of antimicrobial peptides (AMPs) on mycorrhizal associations. AAES Res. Series.

[60] Odom L., Ankumah R.O., Jaynes J., Bonsi C., Cary J.W., Egnin M., Mortley D., Ogden L., Rajasekaran K. (2010). Effect of Transgenic Cotton Plants Transformed with Antimicrobial Synthetic Peptide D4E1 on Cotton Seedling Disease, Soil Microbial Diversity, and Enzymatic Activity, ACS National Meeting.

[61] Shah D.M. (1997). Genetic engineering for fungal and bacterial diseases. Curr. Opin. Biotechnol..

[62] Rajasekaran K., Cary J.W., Jacks T.J., Stromberg K., Cleveland T.E. (2000). Inhibition of fungal growth *in planta* and *in vitro* by transgenic tobacco expressing a bacterial nonheme chloroperoxidase gene. Plant Cell Rep..

[63] Jacks T.J., de Lucca A.J., Rajasekaran K., Stromberg K., van Pee K. (2000). Antifungal and peroxidative activities of nonheme chloroperoxidase in relation to transgenic plant protection. J. Agric. Food Chem..

[64] Sundaresha S., Manoj Kumar A., Rohini S., Math S.A., Keshamma E., Chandrasekhar S.C., Udayakumar M. (2010). Enhanced protection against two major fungal pathogens of groundnut, *Cecospora arachidicola* and *Aspergillus flavus* in transgenic groundnut over-expressing a tobacco â-1-3 glucanase. Eur. J. Plant Pathol..

[65] de Lucca A.J., Jacks T., Broekaert W. (1999). Fungicidal and binding properties of three plant peptides. Mycopathologia.

[66] Fakhoury A.M., Woloshuk C.P. (2001). Inhibition of growth of *Aspergillus flavus* and fungal alpha-amylases by a lectin-like protein from *Lablab purpureus*. Mol. Plant Microbe Interact..

[67] Baker R., Brown R.L., Cleveland T.E., Chen Z.-Y., Fakhoury A. (2009). A maize lectin-like protein with antifungal activity against *Aspergillus flavus*. J. Food Prot..

[68] Nielsen K., Payne G.A., Boston R.S. (2001). Maize ribosome-inactivating protein inhibits normal development of *Aspergillus nidulans* and *Aspergillus flavus*. Mol. Plant Microbe Interact..

[69] Weissinger A., Wu M., Wang X., Isleib T., Stalker T., Shew B., Rajasekaran K., Cary J., Cleveland T.E. Advancement and testing of transgenic peanuts with enhanced resistance to A. flavus. Proceedings of the 2007 Annual Aflatoxin/Fumonisin Workshop.

[70] Cary J.W., Rajasekaran K., Jaynes J.M., Cleveland T.E. (2000). Transgenic expression of a gene encoding a synthetic antimicrobial peptide results in inhibition of fungal growth *in vitro* and *in planta*. Plant Sci..

[71] Rajasekaran K., Cary J.W., Jaynes J.M., Cleveland T.E. (2005). Disease resistance conferred by the expression of a gene encoding a synthetic peptide in transgenic cotton (*Gossypium hirsutum* L.) plants. Plant Biotechnol. J..

[72] Weissinger A., Sampson K., Urban L., Ingram K., Payne G., Scanlon S., Liu Y.S., Cleveland T.E. Transformation with Genes Enoding Peptidyl MIM®,as a Means of Reducing Aflatoxin Contamination in Peanut. Proceedings of the 2000 USDA-ARS Aflatoxin Elimination Workshop.

[73] DeGray G., Rajasekaran K., Smith F., Sanford J., Daniell H. (2001). Expression of an antimicrobial peptide via the chloroplast genome to control phytopathogenic bacteria and fungi. Plant Physiol..

[74] Moore K.G., Price M.S., Boston R.S., Weissinger A.K., Payne G.A. (2004). A chitinase from Tex6 maize kernels inhibits growth of *Aspergillus flavus*. Phytopathology.

[75] Darnetty L.J.F., Muthukrishnan S., Swegle M., Vigers A.J., Selitrennikoff C.P. (1992). Variability in antifungal proteins in the grains of maize, sorghum and wheat. Physiol. Plant..

[76] Wright M.S., Greene-McDowelle D.M., Zeringue H.J., Bhatnagar D., Cleveland T.E. (2000). Effects of volatile aldehydes from *Aspergillus*-resistant varieties of corn on *Aspergillus parasiticus* growth and aflatoxin biosynthesis. Toxicon.

[77] Neucere J.N., Brown R.L., Cleveland T.E. (1995). Correlation of antifungal properties and β-1,3 glucanases in aqueous extracts of kernels from several varieties of corn. J. Agric. Food Chem..

[78] Rajasekaran K., Cary J.W., Chen Z.Y., Brown R.L., Cleveland T.E. (2008). Antifungal traits of a 14 kDa maize kernel trypsin inhibitor protein in transgenic cotton. J. Crop Improv..

[79] Chen Z.Y., Brown R.L., Menkir A., Damann K.E., Cleveland T.E. (2005). Proteome analysis of near isogenic maize lines differing in the level of resistance against *Aspergillus flavus* infection/aflatoxin production. Phytopathology.

[80] Walsh T.A., Morgan A.E., Hey T.D. (1991). Characterization and molecular cloning of a proenzyme form of a ribosome-inactivating protein from maize. Novel mechanism of proenzyme activation by proteolytic removal of a 2.8-kilodalton internal peptide segment. J. Biol. Chem..

[81] Krawetz J.E., Boston R.S. (2000). Substrate specificity of a maize ribosome-inactivating protein differs across diverse taxa. Eur. J. Biochem..

[82] Cordero M.J., Raventos D., San Segundo B. (1992). Induction of PR proteins in germinating maize seeds infected with the fungus *Fusarium moniliforme*. Physiol. Mol. Plant Pathol..

[83] Casacuberta J.M., Raventos D., Puigdomenech P., San Segundo B. (1992). Expression of the gene encoding the PR like protein PRms in germinating maize embryos. Mol. Gen. Genet..

[84] Rajasekaran K., Cary J.W., Jacks T.J., Cleveland T.E., Rajasekaran K., Jacks T.J., Finley J.W. (2002). Genetic Engineering for Resistance to Phytopathogens. Crop Biotechnology.

[85] de Lucca A.J., Cleveland T.E., Wedge D.E. (2005). Plant-derived antifungal proteins and peptides. Can. J. Microbiol..

[86] Broekaert W.F., Cammue B.P.A., de Bolle M.F.C., Thevissen K., de Samblanx G.W., Osborn R.W. (1997). Antimicrobial peptides from plants. Crit. Rev. Plant Sci..

[87] Lin W.C., Lu C.F., Wu J.W., Cheng M.L., Lin Y.M., Yang N.S., Black L., Green S.K., Wang J.F., Cheng C.P. (2004). Transgenic tomato plants expressing the *Arabidopsis NPR1* gene display enhanced resistance to a spectrum of fungal and bacterial diseases. Transgenic Res..

[88] Marcos J.F., Munoz A., Perez-Paya E., Misra S., Lopez-Garcia B. (2008). Identification and rational design of novel antimicrobial peptides for plant protection. Annu. Rev. Phytopathol..

[89] Reddy K.V., Yedery R.D., Aranha C. (2004). Antimicrobial peptides: Premises and promises. Int. J. Antimicrob. Agents.

[90] Blondelle S.E., Lohner K. (2000). Combinatorial libraries: A tool to design antimicrobial and antifungal peptide analogues having lytic specificities for structure-activity relationship studies. Biopolymers.

[91] Rajasekaran K., Jaynes J.M., Cary J.W., Appell M., Kendra D.F., Trucksess M.W. (2009). Transgenic Expression of Lytic Peptides in Food and Feed Crops to Control Phytopathogens and Preharvest Mycotoxin Contamination. Mycotoxin Prevention and Control in Agriculture.

[92] Gao A.G., Hakimi S.M., Mittanck C.A., Wu Y., Woerner B.M., Stark D.M., Shah D.M., Liang J., Rommens C.M. (2000). Fungal pathogen protection in potato by expression of a plant defensin peptide. Nat. Biotechnol..

[93] Rajasekaran K., Cary J.W., Delucca A.J., Jacks T.J., Lax A.R., Cleveland T.E., Chen Z.Y., Chlan C., Jaynes J. *Agrobacterium* Mediated Transformation and Analysis of Cotton Expressing Antifungal Peptides. Proceedings of the 10th USDA ARS Aflatoxin Elimination Workshop.

[94] Rajasekaran K., Stromberg K.D., Cary J.W., Cleveland T.E. (2001). Broad-spectrum antimicrobial activity *in vitro* of the synthetic peptide D4E1. J. Agric. Food Chem..

[95] Florack D.E., Stiekema W.J. (1994). Thionins: Properties, possible biological roles and mechanisms of action. Plant Mol. Biol..

[96] Thomma B.P.H.J., Cammue B.P.A., Thevissen K. (2002). Plant defensins. Planta.

[97] Niu C., Akasaka-Kennedy Y., Faustinelli P., Joshi M., Rajasekaran K., Yang H., Chu Y., Cary J., Ozias-Akins P. (2009). Antifungal activity in transgenic peanut (*Arachis hypogaea* L.) conferred by a nonheme chloroperoxidase gene. Peanut Sci..

[98] Kim Y.H., Woloshuk C.P., Cho E.H., Bae J.M., Song Y.S., Huh G.H. (2007). Cloning and functional expression of the gene encoding an inhibitor against *Aspergillus flavus* alpha-amylase, a novel seed lectin from *Lablab purpureus* (*Dolichos lablab*). Plant Cell Rep..

[99] Tinoco M.L., Dias B.B., Dall’Astta R.C., Pamphile J.A., Aragao F.J. (2010). *In vivo* trans-specific gene silencing in fungal cells by *in planta* expression of a double-stranded RNA. BMC Biol..

